# Balancing High Yield and Metabolic Health in Dairy Ruminants: The Central Hub Role of the Rumen Microbiota

**DOI:** 10.3390/vetsci13060546

**Published:** 2026-06-02

**Authors:** Xingwei Jiang, Xinyi Zhang, Yiyang Sun, Shixi Liu, Xiaodong Chen, Rongzhen Zhong, Yangchun Cao, Qingyu Sun, Shengru Wu

**Affiliations:** 1 College of Animal Science and Technology, Northwest A&F University, Yangling, Xianyang 712100, China; 2 School of Animal Science and Technology, Ningxia University, Yinchuan 750021, China; 3 Jilin Province Feed Processing and Ruminant Precision Breeding Cross Regional Cooperation Technology Innovation Center, Jilin Provincial Laboratory of Grassland Farming, Northeast Institute of Geography and Agroecology, Chinese Academy of Sciences, Changchun 130102, China

**Keywords:** dairy animals, rumen microbiota, periparturient period, subacute ruminal acidosis, ketosis, rumen epithelium, VFA

## Abstract

Modern dairy production has greatly increased milk yield, but high productivity is often accompanied by greater metabolic pressure, particularly during the transition period. Ketosis, fatty liver, and subacute ruminal acidosis are major disorders that limit health, efficiency, and sustainability in high-yielding dairy ruminants. This review examines the rumen microbiota as a central biological interface linking diet, ruminal fermentation, epithelial function, hepatic metabolism, and inflammation. Under homeostatic conditions, the rumen microbiota supports lactation by converting dietary fibre, starch, and nitrogen into volatile fatty acids, microbial protein, and other metabolites required for gluconeogenesis, milk component synthesis, and epithelial maintenance. However, under excessive nutritional or physiological stress, especially high-concentrate feeding and periparturient negative energy balance, this system may shift toward dysbiosis, acid accumulation, lipopolysaccharide release, epithelial barrier impairment, and activation of gut–liver inflammatory pathways. These changes can contribute to the occurrence and interaction of subacute ruminal acidosis, ketosis, and fatty liver. We further summarize key factors affecting rumen microbial stability, including diet structure, host variation, physiological stage, environmental stress, feeding management, and ruminal epithelial volatile fatty acid absorption. Finally, microbiome-oriented strategies, such as gradual dietary transition, nutritional preconditioning, probiotics, postbiotics, functional metabolites, host metabolic support, and epithelial-targeted interventions, are discussed. Maintaining rumen microbial homeostasis should be regarded as a core principle for balancing high milk yield with long-term metabolic health. Future research should move beyond descriptive profiling toward causal validation of host–microbe interactions and the development of microbiome-based early-warning and individualized nutritional management systems.

## Simple Summary

High milk yield in dairy ruminants depends on intensive nutrient conversion, but this process can increase the risk of metabolic disorders when rumen and host adaptation are not well coordinated. This review highlights the rumen microbiota as a central hub linking diet, ruminal fermentation, epithelial barrier function, liver metabolism, and inflammation. The main insight is that rumen microbes have a dual role: under stable conditions, they convert feed into volatile fatty acids and microbial protein that support milk production; under high-concentrate feeding, abrupt dietary transition, or periparturient stress, microbial imbalance may promote acid accumulation, inflammatory signals, epithelial damage, and greater risk of subacute ruminal acidosis, ketosis, and fatty liver. These findings benefit dairy scientists by providing an integrated framework for studying host–microbe mechanisms, and benefit nutritionists, veterinarians, and producers by emphasizing microbial stability as a practical target for transition management and disease prevention. Future applications may include microbiome-based early-warning tools, individualized feeding strategies, and interventions that support rumen epithelial absorption, metabolic resilience, animal welfare, and sustainable milk production.

## 1. Introduction

Over the past several decades, genetic selection, refined diet formulation, and intensive management have substantially increased milk yield in modern dairy production systems [1]. However, this improvement in productivity has been accompanied by persistent metabolic pressure, particularly during the transition from late pregnancy to early lactation. During this period, high-yielding dairy ruminants must rapidly redirect nutrients toward milk fat, milk protein, and lactose synthesis while maintaining energy balance, immune homeostasis, rumen function, and hepatic metabolism [2,3]. This creates a central paradox in modern dairy production: the same biological and nutritional conditions required to support high milk yield may increase the risk of metabolic disorders when host adaptive capacity is exceeded.

The global burden of dairy cow diseases further highlights the practical importance of this problem. A recent comorbidity-adjusted economic analysis estimated that major dairy cattle diseases cause approximately US$65 billion in annual global losses, with subclinical ketosis alone accounting for about US$18 billion, making it one of the costliest disorders in dairy production systems [4]. Consistently, a large multi-regional field study covering Central and South America, Africa, Asia, Australia, New Zealand, and Eastern Europe reported that subclinical ketosis is widespread in early-lactation dairy cows, with prevalence varying markedly among regions and production systems [5]. These data indicate that metabolic disorders are not local or occasional events, but global constraints on productivity, profitability, animal welfare, and sustainable dairy production. Therefore, understanding the biological mechanisms that link high milk yield with metabolic disease susceptibility is essential for developing effective prevention and precision management strategies.

Ketosis, fatty liver, and subacute ruminal acidosis (SARA) are among the most important metabolic constraints affecting high-yielding dairy cows. Ketosis and fatty liver are closely associated with negative energy balance, excessive lipid mobilization, elevated non-esterified fatty acids and β-hydroxybutyrate, and hepatic lipid overload during early lactation [3,6,7]. SARA, in contrast, is generally regarded as a ruminal fermentation disorder driven by high intake of rumen-degradable starch (RDS), insufficient physically effective fibre, impaired ruminal buffering, and prolonged depression of ruminal pH [8,9]. Although these diseases are often discussed as separate conditions, they frequently converge in high-producing animals through shared biological processes, including altered ruminal fermentation, epithelial barrier dysfunction, endotoxin-related inflammation, immune dysregulation, and hepatic metabolic stress [9,10,11,12]. Therefore, the challenge in high-yielding dairy production is not simply to increase nutrient supply, but to maintain metabolic resilience under high nutritional input.

Traditional research has explained these disorders mainly from the perspectives of dietary energy density, nutrient supply–demand imbalance, endocrine adaptation, hepatic lipid metabolism, and immune suppression. These mechanisms remain essential, but they do not fully explain why animals exposed to similar diets and production pressures show markedly different metabolic outcomes. In recent years, advances in metagenomics, metabolomics, transcriptomics, and integrated multi-omics have shifted attention toward the rumen microbiota as a key biological interface linking diet, fermentation, epithelial absorption, host metabolism, and inflammatory responses [13,14,15]. The rumen microbiota converts dietary substrates into volatile fatty acids (VFAs), microbial protein, and other metabolites that sustain gluconeogenesis, milk component synthesis, and nitrogen utilization [13,14,16]. However, the metabolic value of this fermentation system depends not only on microbial production, but also on its functional coupling with the rumen epithelium, which determines the efficiency of nutrient absorption, acid clearance, and barrier maintenance. When this coupling is disrupted by high-concentrate feeding, abrupt dietary transition, periparturient stress, or impaired epithelial adaptation, the same microbial ecosystem may shift from a productivity-supporting state to a disease-promoting state. Microbial dysbiosis can accelerate acid accumulation, increase lipopolysaccharide and other fermentation-derived inflammatory stimuli, impair epithelial integrity, and increase the inflammatory and metabolic burden on the liver [12,17,18]. Thus, the rumen microbiota should not be viewed merely as a digestive microbial community or a passive indicator of dietary change, but as a central metabolic–inflammatory interface that determines whether high nutrient input is converted into productive substrates or redirected toward pathological pathways.

Despite increasing evidence linking rumen microbiota to metabolic health, the current literature remains fragmented. Many studies have described microbial compositional changes associated with SARA, ketosis, fatty liver, or periparturient metabolic stress, but fewer have integrated these findings into a unified framework explaining how microbial function, epithelial absorption, hepatic metabolism, and inflammation interact to determine the balance between high productivity and health [17,19,20,21]. Recent studies provide empirical support for this microbiota–epithelium–host framework. In dairy cows, animals differing in SARA susceptibility show distinct rumen microbiome and metabolome responses under similar acidogenic challenges, suggesting that disease risk reflects individual microbial and metabolic adaptability rather than diet composition alone [19]. Multi-omics analysis in lactating dairy cows further revealed coordinated alterations in the rumen microbiome and rumen epithelium during high-grain-induced SARA, indicating that microbial dysbiosis and epithelial responses are closely coupled [18]. Mechanistic studies in dairy goats have extended this concept by showing that SARA tolerance is associated with efficient VFA absorption, epithelial proliferation, microbial functional stability, and restrained inflammatory responses under high-concentrate challenge [20,21,22,23,24].

In this review, we propose that the rumen microbiota functions as a central regulatory hub linking high productivity with metabolic health in dairy ruminants. Rather than treating SARA, ketosis, and fatty liver as isolated disorders, we examine them within a unified microbiota–epithelium–liver–inflammation framework. This review focuses on the dual role of the rumen microbiota in supporting nutrient conversion and amplifying metabolic disease risk, and further discusses microbiome-oriented precision regulation strategies for balancing high milk yield with long-term metabolic health.

## 2. Metabolic Adaptation and Disease Susceptibility in High-Yielding Dairy Ruminants

### 2.1. Periparturient Negative Energy Balance as a Challenge to Metabolic Resilience

The transition period is a critical window in which high-yielding dairy cows must shift from pregnancy maintenance to intensive milk synthesis within a short time. After calving, the demand for glucose, fatty acids, and amino acids increases rapidly to support lactose, milk fat, and milk protein synthesis, whereas dry matter intake often recovers more slowly. This mismatch results in negative energy balance (NEB), which is a physiological adaptation to early lactation but becomes pathological when its magnitude or duration exceeds the cow’s metabolic capacity [2,3].

The primary consequence of severe or prolonged NEB is excessive adipose tissue mobilization. Increased release of non-esterified fatty acids (NEFA) provides an alternative energy source for peripheral tissues and the liver; however, when hepatic uptake of NEFA exceeds the capacity for complete oxidation or export as very-low-density lipoproteins, triglycerides accumulate in the liver and ketone body production increases. This metabolic sequence links early-lactation NEB with fatty liver and ketosis, two closely related disorders that reflect impaired coordination between lipid mobilization, hepatic oxidation, gluconeogenesis, and ketogenesis [3,6,10].

Therefore, NEB should not be interpreted simply as an energy deficit. It represents a systemic redistribution of nutrients and metabolic priorities during the onset of lactation. When this redistribution remains within the adaptive range, it supports milk production; when it is excessive, it increases hepatic lipid burden, ketone body accumulation, oxidative stress, and inflammatory susceptibility.

### 2.2. Fermentable Carbohydrate Pressure and Ruminal Acid-Load Adaptation

Diets for high-yielding dairy animals increase energy supply during the periparturient and lactating periods by raising the concentrate ratio and starch levels. Although this strategy increases energy density, it also increases acid pressure in the rumen. When large amounts of readily fermentable carbohydrates enter the rumen, they are rapidly degraded by microbes to produce VFA and lactic acid, under some conditions, lactate. SARA develops when acid production exceeds the combined capacity of salivary buffering, ruminal passage, microbial acid utilization, and epithelial absorption, resulting in a prolonged depression of ruminal pH [9,25]. However, SARA should not be interpreted as a simple linear consequence of high-concentrate feeding or a single pH threshold. Rather, it reflects a dynamic imbalance among fermentable substrate supply, acid production, buffering capacity, epithelial absorption, and microbial adaptation [9,25]. This view explains why animals exposed to similar acidogenic diets may show different degrees of pH depression, inflammatory response, and production loss. Real-time monitoring and multi-omics studies further indicate that SARA is accompanied by microbial community restructuring, metabolic pathway remodelling, and epithelial responses, suggesting that individual tolerance depends on the coordination between microbial fermentation and host epithelial adaptation [17,23,26,27]. More accurately, high-concentrate feeding increases the acid load and the risk of SARA when the supply rate of fermentable carbohydrates exceeds the adaptive capacity of the rumen ecosystem and host tissues. Under conditions of a smooth dietary transition, the rumen microbiota, rumen epithelium, salivary buffering, VFA absorption, and microbial acid-utilizing populations may gradually adapt to the higher supply of fermentable substrates, enabling some cows to tolerate relatively high concentrate levels without developing severe ruminal dysfunction. Therefore, SARA should be understood more as a failure of adaptation rather than a simple inevitable consequence of increased concentrate proportions. When large amounts of readily fermentable carbohydrates enter the rumen abruptly, or when their supply is not matched with sufficient physically effective fibre and acid-clearance capacity, VFA and, in some cases, lactate may accumulate. SARA occurs when acid production exceeds the combined capacity of salivary buffering, ruminal passage, microbial acid utilization, and epithelial absorption, manifesting as a prolonged depression of ruminal pH and a disruption of microbial-epithelial homeostasis.

The pathological significance of SARA lies in its chronic and recurrent nature. Even in the absence of acute clinical signs, repeated ruminal pH depression can impair fibre digestion, reduce milk fat synthesis, increase ruminal endotoxin load, weaken epithelial barrier function, and promote local or systemic inflammatory responses [8,28,29]. Therefore, SARA represents not only a disorder of ruminal fermentation but also a failure of the rumen ecosystem to maintain acid clearance, epithelial integrity, and inflammatory control under high nutritional input.

### 2.3. Convergent Metabolic and Inflammatory Pathways Among Ruminal and Hepatic Disorders

Ketosis and fatty liver are usually classified as energy metabolism disorders, whereas SARA is generally considered a ruminal fermentation disorder. This distinction is useful for diagnosis, but it can obscure the biological overlap among these conditions in high-yielding dairy cows. During the periparturient period, excessive lipid mobilization increases hepatic NEFA influx and ketone body production, while high-concentrate feeding increases ruminal acid load and microbial inflammatory stimuli. These two pathological pressures may converge at the level of hepatic metabolism and systemic inflammation [9,10,11]. On the ruminal side, SARA-associated dysbiosis and epithelial barrier dysfunction can increase the exposure of the host to lipopolysaccharide (LPS) and other fermentation-derived inflammatory stimuli, thereby adding an inflammatory burden to the liver and peripheral tissues [28,29]. On the metabolic side, fatty liver and ketosis reflect impaired hepatic handling of NEFA, triglyceride accumulation, and ketogenesis; these changes may reduce hepatic metabolic flexibility and increase susceptibility to inflammatory stress during early lactation [3,6,10]. Recent microbiome studies further suggest that rumen and hindgut microbial disturbances are associated with postpartum energy metabolism disorders, indicating that gastrointestinal microbial signals may participate in the progression from metabolic adaptation to disease [20,21]. Thus, ketosis, fatty liver, and SARA should not be viewed as completely independent disorders. NEB provides the lipid-mobilization and ketogenesis pressure, high-concentrate feeding provides the ruminal acid and endotoxin challenge, and inflammation links these local disturbances into a broader metabolic disease network. This integrated view is important because it shifts the focus from single-disease management toward maintaining coordinated function among ruminal fermentation, epithelial barrier integrity, hepatic metabolism, and immune regulation.

### 2.4. From Ruminal Imbalance to Systemic Metabolic and Immune Consequences

In SARA, prolonged ruminal pH depression and increased endotoxin production can increase the risk of inflammatory complications [8,30]. Acidosis has also been linked to laminitis and lameness, although this relationship is not driven by ruminal pH alone; systemic inflammation, endotoxin exposure, altered vascular function, and hoof tissue hypoperfusion may all contribute to the development of claw lesions [30,31]. Similarly, NEB-associated lipid mobilization and fatty liver can impair immune function during the transition period. Elevated NEFA and β-hydroxybutyrate, oxidative stress, and inflammatory activation may compromise neutrophil function and other innate immune responses, thereby increasing susceptibility to mastitis, uterine disease, and other periparturient disorders [10,11,32]. These systemic effects explain why metabolic disorders in high-yielding dairy cows often manifest not as isolated biochemical abnormalities, but as combined reductions in production efficiency, immune competence, reproductive performance, and productive lifespan.

Therefore, the pathological features of high-yield-associated metabolic diseases are best understood as a progression from local metabolic imbalance to systemic homeostatic disruption. NEB, SARA, ketosis, and fatty liver arise from different initiating pressures, but they interact through shared inflammatory and metabolic pathways. This provides the physiological basis for considering the rumen microbiota as a key interface connecting nutritional input, ruminal fermentation, epithelial integrity, hepatic metabolism, and immune regulation.

## 3. Rumen Microbiota as a Regulator of Nutrient Partitioning and Lactation Support

### 3.1. Rumen Microbiota as a Vital Metabolic Organ in High-Yielding Ruminants

The rumen microbiota, composed of bacteria, archaea, protozoa, anaerobic fungi, and viruses, constitutes a complex fermentation ecosystem that enables ruminants to utilize feed resources that cannot be efficiently digested by the host itself [13]. Through microbial fermentation, plant fibre, starch, soluble carbohydrates, and non-protein nitrogen are converted into volatile fatty acids (VFAs), microbial protein (MCP), and other bioactive metabolites, thereby forming the biochemical basis of ruminant productivity [13,16,33]. In this sense, the rumen microbiota acts as an “invisible engine” behind high milk production: it does not produce milk directly, but determines how efficiently dietary substrates are transformed into absorbable energy and protein resources for lactation.

### 3.2. Propionate: The Gluconeogenic Foundation for High-Yielding Lactation

Among the major VFAs, propionate has a unique role because it is the principal ruminal precursor for hepatic gluconeogenesis in ruminants [33,34]. This is particularly important for high-yielding dairy cows because glucose supply is required for lactose synthesis, and lactose plays a major role in regulating milk volume through its osmotic effect in the mammary gland [35,36]. Therefore, the contribution of propionate to high productivity should be understood as a microbial route through which ruminal fermentation supports glucose availability and milk yield.

### 3.3. Acetate and Butyrate: Sources for Milk Fat Synthesis and Epithelial Nutrition

Acetate and butyrate represent another major route by which rumen microbiota support lactation. Acetate is an important substrate for de novo fatty acid synthesis in the mammary gland and is closely related to milk fat production, whereas butyrate is extensively metabolized by the rumen epithelium and contributes to epithelial growth and functional maturation [14,33,37,38]. Therefore, the value of ruminal fermentation cannot be assessed only by total VFA concentration; the molar profile and balance among acetate, propionate, and butyrate determine whether fermentation products are more directed toward milk volume, milk fat synthesis, or epithelial development. This distinction is important for high-yielding dairy cows because high milk output requires not only abundant fermentation end products, but also an appropriate VFA pattern. Diets that excessively shift fermentation toward propionate at the expense of acetate may support glucogenic supply but can also reduce the acetate-to-propionate ratio and increase the risk of milk fat depression under some dietary conditions [37,38,39]. Thus, productive rumen fermentation is not equivalent to maximal acid production; it requires a coordinated VFA profile that matches the metabolic demands of lactation.

### 3.4. Microbial Protein: A Crucial Amino Acid Reservoir in Early High-Yielding Lactation

Beyond energy supply, the rumen microbiota contributes substantially to protein nutrition. Ruminal microbes incorporate ammonia, peptides, amino acids, and non-protein nitrogen into MCP, which flows to the small intestine and provides a high-quality source of absorbable amino acids for the host [16]. This microbial protein is especially important in high-yielding dairy cows because milk protein synthesis depends on a stable supply of metabolizable amino acids rather than dietary crude protein intake alone [16,40]. MCP should therefore be viewed as a biological upgrading system for dietary nitrogen. Through microbial growth, nitrogen sources of variable quality can be transformed into a more balanced amino acid supply for milk protein synthesis and tissue metabolism. The contribution of MCP varies with diet composition, rumen-degradable protein supply, fermentable energy availability, and microbial growth efficiency, but it generally represents a major component of metabolizable protein supply in dairy cows [16,41]. Recent microbiome-based studies further suggest that specific rumen microbes involved in amino acid biosynthesis, such as *Ruminococcus_E bovis*, may contribute to glucogenic amino acid supply and postpartum energy metabolism, although such mechanisms require further validation across diets and production systems [42].

### 3.5. Rumen Epithelial VFA Absorption: A Crucial Channel Connecting Rumen Microbiota and the Host

Taken together, a healthy rumen microbiota is not merely a microbial community present in the digestive tract; it is a functional conversion system that determines how dietary fibre, starch, and nitrogen are transformed into milk-supporting substrates. Propionate primarily supports glucose and lactose synthesis, acetate and butyrate contribute to milk fat synthesis and rumen development, and microbial protein MCP supplies absorbable amino acids for milk protein production [14,16,33,37]. Therefore, the productivity-supporting capacity of the rumen microbiota depends on the coordinated generation of energy substrates and amino acid resources, rather than on the abundance of any single microbial taxon or metabolite. This perspective provides the basis for understanding why rumen microbial stability is central to high-yielding dairy production. When microbial fermentation remains functionally balanced, the rumen acts as an efficient feed-to-milk conversion platform. When this balance is disrupted, the same high-input feeding system may lose efficiency and become vulnerable to metabolic disturbances. Thus, the functional integrity of the rumen microbiota is a cornerstone of high productivity and a prerequisite for maintaining metabolic health in dairy ruminants [13,15].

## 4. Microbial Signals in the Transition from Adaptation to Metabolic Disease

### 4.1. Microbial Imbalance Under High-Concentration Pressure

**Figure 1:**
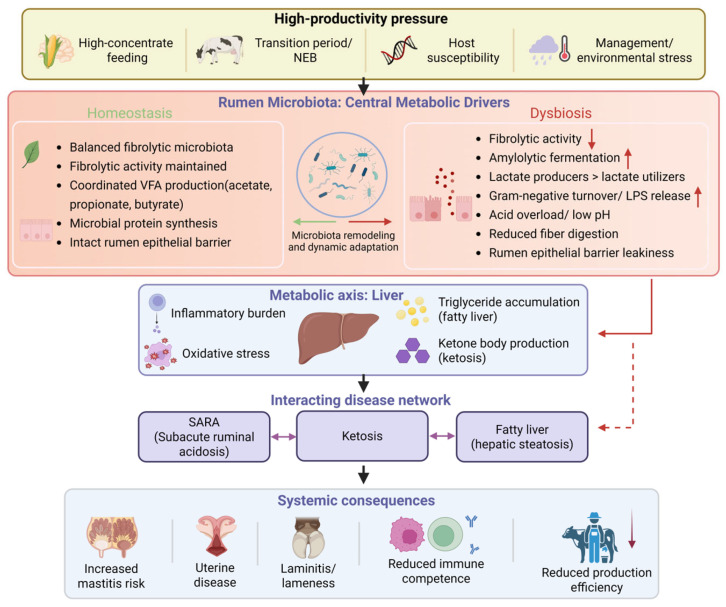
The microbiota–metabolism–disease axis in high-yielding dairy ruminants. High-productivity pressure, including high-concentrate feeding, transition period-associated negative energy balance, host susceptibility, and environmental stress, can shift the rumen microbiota from a homeostatic state to dysbiosis. Homeostatic microbiota support fibre degradation, coordinated VFA production, microbial protein synthesis, and epithelial barrier integrity, whereas dysbiosis is characterized by reduced fibrolytic activity, enhanced amylolytic fermentation, lactate imbalance, LPS release, acid overload, and barrier leakiness. These microbial changes converge on the liver through inflammatory and oxidative stress pathways, promoting triglyceride accumulation, ketone body production, and the interaction among SARA, ketosis, and fatty liver, with systemic consequences including mastitis risk, uterine disease, laminitis or lameness, reduced immune competence, and lower production efficiency. VFA, volatile fatty acid; LPS, lipopolysaccharide; SARA, subacute ruminal acidosis; NEB, negative energy balance.

High-concentrate feeding is widely used to increase dietary energy density in high-yielding dairy cows, but it also imposes strong ecological pressure on the rumen microbiota. When readily fermentable carbohydrates replace a large proportion of structural fibre, the rumen ecosystem tends to shift from a fibre-degrading mode toward a rapid-fermentation mode. This transition is typically characterized by reduced activity of pH-sensitive fibrolytic populations and increased dominance of starch- and sugar-fermenting bacteria, thereby changing both fermentation pattern and microbial interaction networks [9,43]. This shift should not be interpreted as a simple increase or decrease in a few microbial taxa. Real-time monitoring during SARA development has shown that the rumen microbiota undergoes a staged and dynamic restructuring process, in which microbial interactions and functional pathways change continuously as acidogenic pressure increases [17]. Studies in dairy cows further suggest that individual susceptibility to SARA is associated with differences in rumen metabolic environment and microbial fermentative responses, indicating that disease risk reflects not only diet composition but also the adaptive capacity of the microbial ecosystem [26,27]. Therefore, the starting point of SARA is not high-concentrate feeding alone, but the failure of the rumen microbiota to maintain functional balance under high substrate pressure (Figure 1).

### 4.2. Lactate Accumulation and pH Decline Induced by Rumen Fermentation Disturbance

Under high-concentration conditions, rapidly fermentable carbohydrates are converted into VFAs and, under some circumstances, lactate. Moderate acid production is necessary for energy supply, but excessive or poorly coordinated acid production causes ruminal pH depression. Lactate accumulation is most likely to occur when lactate-producing bacteria outpace lactate-utilizing bacteria, whereas VFA accumulation and insufficient acid removal are major contributors to SARA in lactating dairy cows [44]. The decline in ruminal pH further suppresses pH-sensitive fibrolytic bacteria and reduces fibre degradation, which reinforces the shift away from stable fibre fermentation. In this sense, SARA is a self-reinforcing ecological and metabolic imbalance: rapid fermentation increases acid load, low pH weakens fibre-degrading function, and reduced fibre digestion further destabilizes rumen fermentation [8]. Thus, high fermentative capacity is beneficial only when it remains coordinated with ruminal acid clearance.

### 4.3. Increased LPS and Rumen Inflammation

As ruminal pH declines, the growth, death, and lysis patterns of Gram-negative bacteria may change, increasing the release of lipopolysaccharide (LPS) into rumen fluid. Grain-induced SARA in dairy cows has been shown to increase ruminal LPS concentration and activate inflammatory responses, supporting the view that microbial products are central mediators linking ruminal dysbiosis with host inflammation. LPS can interact with pattern-recognition pathways and contribute to epithelial inflammatory activation, barrier dysfunction, and systemic immune responses when mucosal integrity is compromised [28,29]. However, microbial metabolites should not be classified simply as harmful by-products. Their effects depend on the metabolic context, host response, and barrier status. For example, altered ruminal tryptophan metabolism in SARA-affected dairy goats can generate 3-indoleacetic acid, which has been shown to inhibit Th17/IL-17-related inflammation and alleviate ruminal epithelial injury [22]. This finding indicates that microbial metabolites may either amplify inflammation or contribute to host protection, depending on their chemical identity and the local microenvironment.

### 4.4. Barrier Disruption and Gut-Liver Axis Activation

The transition from local ruminal disturbance to systemic metabolic disorder depends largely on barrier integrity. Prolonged low pH, increased toxin load, and inflammatory stimulation can reduce the barrier function of the rumen and intestinal epithelia, increasing the possibility that LPS and other microbial products enter the portal circulation [29]. Once these signals reach the liver, they add an inflammatory burden to an organ already responsible for nutrient partitioning, detoxification, gluconeogenesis, and lipid metabolism during early lactation [3,10]. This gut–liver connection is not restricted to ruminal LPS. Recent microbiome studies suggest that hindgut microbial imbalance can aggravate postpartum energy metabolism disorders by reducing acetate-mediated hepatic AMPK–PPARA signalling, whereas ileal microbiota and secondary bile acids can influence susceptibility to nonalcoholic steatohepatitis in dairy goats through hepatic Treg/Th17 immune balance [20,45]. These studies broaden the traditional SARA-centred view by showing that microbial signals from different gastrointestinal segments may converge on hepatic metabolism and immune regulation.

### 4.5. Inflammation and NEB Form a Vicious Cycle

Persistent inflammation can amplify the metabolic burden of high-yielding cows. Inflammatory activation is energetically costly and can impair insulin sensitivity, promote lipid mobilization, and increase hepatic metabolic pressure. During the transition period, this may intensify NEFA influx into the liver, impair lipid handling, and favour triglyceride accumulation and ketone body production [3,10,11]. Therefore, inflammation does not merely accompany metabolic disease; it can help convert an adaptive energy deficit into a pathological state. This feedback also explains why SARA, ketosis, and fatty liver often interact rather than occur as independent disorders. SARA-associated microbial products can increase inflammatory load, whereas fatty liver and ketosis may reduce hepatic metabolic flexibility and immune competence. As a result, microbial dysbiosis, inflammation, lipid mobilization, and hepatic dysfunction may reinforce one another during early lactation [10,20,21]. The systemic consequences include reduced feed efficiency, impaired immune defence, increased susceptibility to mastitis and uterine disease, and greater risk of hoof disorders such as laminitis and lameness. The association between acidosis and laminitis is complex, but systemic inflammation, endotoxin exposure, vascular dysfunction, and hoof tissue hypoperfusion are considered important contributors [30,31]. Thus, the nature of rumen microbiota lies in its capacity to support high nutrient conversion under homeostasis while driving inflammatory and metabolic amplification when ecological balance and barrier protection fail. Taken together, microbial metabolites play context-dependent roles in dairy ruminants, either supporting high productivity or driving metabolic imbalance. As illustrated in Table 1, specific microbial metabolites can act either as productivity-supporting substrates or as drivers of metabolic imbalance, depending on environmental and dietary context.

## 5. Determinants of Rumen Microbiota-Mediated Metabolic Resilience

### 5.1. Nutritional Factors

Dietary composition is the most direct and powerful determinant of rumen microbiota stability. The forage-to-concentrate ratio, physically effective neutral detergent fibre (peNDF), starch source, ruminal degradability, fat source, particle size, and feeding frequency collectively shape substrate availability, ruminal retention time, chewing activity, saliva secretion, and fermentation pattern [38,47]. Among these factors, the balance between peNDF and ruminally degradable starch is particularly important because it determines whether the rumen ecosystem is maintained in a fibre-fermenting, pH-stable state or pushed toward rapid acid production [25,47]. Therefore, nutritional effects on the rumen microbiota should be understood not only as changes in nutrient supply, but also as regulation of fermentation kinetics and microbial ecological pressure.

### 5.2. Host Factors: Genetic Background, Physiological Stage, and Individual Variation

Although diet provides the substrate, the host partly determines which microbial communities can colonize, persist, and express their functions. Host genotype, rumen epithelial phenotype, immune status, parity, and lactation stage can influence rumen microbial composition and metabolic activity [14,48]. Studies in cattle have shown that some rumen microbial features are heritable and associated with feed efficiency, indicating that host genetic background can contribute to individual differences in rumen microbiota structure and function [48]. This host effect is particularly important for explaining why animals exposed to similar diets do not show identical rumen responses. Cows or goats facing comparable acidogenic challenges may differ in ruminal pH dynamics, microbial metabolic pathways, epithelial adaptation, and inflammatory responses, resulting in different degrees of SARA susceptibility or tolerance [23,26,49]. Thus, rumen microbiota stability is an outcome of host selection pressure and host–microbe compatibility.

### 5.3. Dramatic Physiological Changes During the Periparturient Period

The periparturient period is a vulnerable window for rumen and intestinal microbiota stability because feed intake, diet composition, endocrine status, immune function, and energy metabolism all change rapidly around calving [50]. During this period, the microbiota must adapt simultaneously to altered substrate supply and host physiological remodelling, which increases the probability of unstable fermentation and metabolic disorder [21,51]. Recent studies suggest that microbial dynamics during the transition period are associated with subsequent lactation performance and disease risk, including postpartum ketosis and energy metabolism disorders [20,21]. Therefore, the periparturient period should be regarded not only as a metabolic risk phase, but also as a critical window for microbial monitoring and early nutritional intervention.

### 5.4. Environmental and Management Factors

Environmental and management factors influence rumen microbiota mainly by modifying feeding behaviour, stress physiology, and hygiene-related exposure. Heat stress, stocking density, feeding competition, bedding hygiene, feed delivery pattern, and diet transition speed can alter dry matter intake, meal size, feeding rhythm, rumination time, and immune status, thereby indirectly reshaping rumen fermentation and microbial stability [8,52]. Heat stress is a representative example: recent evidence shows that heat-stressed Holstein and Jersey cows exhibit marked changes in rumen microbial composition and metabolite profiles, accompanied by oxidative and inflammatory responses [53]. These factors are often simplified or controlled in experimental designs, but they are highly relevant in commercial dairy systems. A diet that is theoretically balanced may still become acidogenic when cows experience heat stress, irregular feed access, intense competition, or abrupt ration changes. Therefore, maintaining rumen microbiota stability requires not only appropriate diet formulation but also management practices that support consistent intake, rumination, and adaptation.

### 5.5. Ruminal Epithelial VFA Absorption Function

Rumen microbiota stability should not be defined solely by stable microbial composition. A more functional definition should include whether microbial fermentation products can be efficiently removed from the rumen and used by the host. In this context, ruminal epithelial VFA absorption is an important host-side stabilizer because it contributes to acid clearance and helps prevent excessive accumulation of fermentation acids [9,14]. When rumen epithelial transport, proliferation, and barrier functions are maintained, the animal is better able to tolerate fluctuations in acid production under high-concentrate feeding. Conversely, impaired epithelial function may reduce the capacity to cope with ruminal acid load and increase susceptibility to SARA [23]. Recent studies in dairy goats further suggest that rumen fungal thiamine metabolism can promote epithelial cell proliferation through the IGFBP2/IGF1 axis, thereby supporting VFA absorption and SARA tolerance [24]. Therefore, epithelial VFA absorption capacity should be considered a core host indicator for evaluating rumen ecosystem stability and the balance between high productivity and metabolic health.

## 6. Microbiome-Oriented Strategies for Improving Metabolic Resilience

### 6.1. Nutritional Preconditioning and Smooth Transition

For high-yielding dairy cows, the first principle of rumen microbiome regulation is prevention rather than treatment. The rumen microbiota requires time to adapt to changes in fermentable substrate supply; therefore, abrupt increases in concentrate proportion or ruminally degradable starch can destabilize microbial fermentation before the host has developed sufficient buffering, absorptive, and metabolic capacity. Gradual dietary transition during the dry period and early lactation, together with adequate peNDF, controlled starch degradability, and consistent feeding rhythm, provides a more stable ecological environment for microbial adaptation and reduces the risk of SARA-related fermentation disorder [2,9,25]. The key point is that nutritional preconditioning should not be understood simply as lowering dietary energy density. High-yielding cows still require a sufficient energy supply, but the rate and synchrony of substrate fermentation need to be controlled. In this sense, diet formulation should aim to match ruminally degradable starch with effective fibre, chewing activity, passage rate, and microbial adaptation, rather than pursuing maximal fermentable carbohydrate supply. Such a strategy allows the rumen microbiota to remain productive without shifting toward acid accumulation and inflammatory stress [47].

### 6.2. Probiotics, Postbiotics, and Microbiota Restoration

Direct microbial regulation aims to reshape fermentation pathways, enhance microbial resilience, and reduce the accumulation of harmful metabolites. Probiotics targeting lactate metabolism are one of the most intuitive approaches. Lactate-utilizing bacteria such as *Megasphaera elsdenii* have been proposed as candidates for reducing ruminal lactate accumulation and improving adaptation to high-grain feeding, although their efficacy may depend on strain, dose, diet composition, and production stage [44,54,55].

Yeast-based products and postbiotics represent another practical strategy for improving rumen microbial stability. Rather than simply increasing the abundance of one beneficial microorganism, yeast fermentation-derived postbiotics may stabilize rumen microbial networks and increase the diversity or connectivity of hub taxa during grain-based SARA challenges in lactating dairy cows [56]. Native rumen microbial supplements have also been reported to improve lactation performance and feed efficiency, suggesting that restoration of functional microbial consortia may be more relevant than single-taxon manipulation in complex rumen ecosystems [57]. Therefore, these additives should be evaluated as fermentation modulators rather than universal SARA-prevention tools.

Phage-based strategies have been proposed as a potential route for precision modulation of rumen microbial populations, because phages can selectively lyse bacterial hosts and thereby influence microbial community structure and function [58,59]. However, their application for preventing SARA or improving dairy cow metabolic health remains largely conceptual, owing to the complexity of rumen microbial networks, narrow host specificity of phages, and limited in vivo validation in production systems [58,59,60].

### 6.3. Ionophore-Based Modulation of Rumen Fermentation During the Transition Period

Ionophore-based interventions represent another commercially relevant strategy for regulating rumen fermentation during the transition period. Among them, monensin has been widely used to modify rumen microbial activity and improve energy metabolism in dairy cattle [61]. Unlike probiotics or postbiotics, which aim to support or restore beneficial microbial networks, monensin acts primarily by selectively inhibiting ion-sensitive bacterial populations and shifting ruminal fermentation toward greater propionate production and a lower acetate-to-propionate ratio [62]. This change is nutritionally important because propionate is the major gluconeogenic precursor in ruminants and can help support hepatic glucose production during early lactation [46]. Systematic evidence indicates that monensin delivered as a controlled-release capsule during the transition period can increase ruminal propionate production, reduce blood β-hydroxybutyrate concentration, and lower the risk of ketosis in dairy cows, although the magnitude of response varies across herds, diets, and management conditions [63]. Therefore, ionophore-based strategies can be viewed as microbiome-directed tools that improve energy efficiency by redirecting ruminal fermentation pathways.

From the perspective of rumen microbial ecology, monensin does not simply increase or decrease total microbial abundance; rather, it reshapes the microbial spectrum and fermentation network. Its antimicrobial activity is generally stronger against Gram-positive and ionophore-sensitive bacteria, which can reduce lactate-producing and hydrogen-producing populations, decrease methane-related hydrogen loss, and favour bacteria and pathways associated with propionate formation [61,62]. These effects may improve fermentation efficiency and reduce ketogenesis risk by increasing glucogenic substrate supply. In theory, reduced lactate accumulation and improved fermentation efficiency may also help buffer acidogenic pressure under some feeding conditions. However, monensin should not be considered a universal strategy for preventing SARA. SARA susceptibility is determined by the balance among fermentable carbohydrate supply, effective fibre, ruminal buffering, epithelial VFA absorption, and microbial adaptation. Therefore, ionophores may support ruminal adaptation but cannot replace appropriate dietary transition, peNDF supply, or feeding management.

In addition, due to its selective antimicrobial pressure, monensin may alter microbial diversity, reduce certain cellulolytic bacteria or non-target microbial populations, and reshape microbial interaction networks. These shifts could potentially limit fibre degradation by reducing overall microbial diversity. Furthermore, given the varying regulatory frameworks across different countries and regions, the use of monensin should be approached with caution. Therefore, while ionophore interventions can enhance production efficiency by modulating the microbiome, their application requires strict adherence to local regulations and a careful weighing of the potential benefits against the risks.

### 6.4. Indirect Regulation of Host–Microbe Interactions

Not all precision strategies need to act directly on rumen microbes. Some interventions may protect metabolic health by reducing the host-side consequences of microbial instability. During the transition period, rumen-protected nutrients such as choline, methionine, selected amino acids, or glucogenic precursors can support hepatic lipid metabolism, antioxidant capacity, immune function, and milk synthesis, thereby reducing the metabolic pressure associated with NEB and excessive fat mobilization [3,32,64]. These strategies do not rebuild the rumen microbiota directly, but they may improve the host’s capacity to tolerate microbial and metabolic fluctuations.

Another host-oriented target is the mucosal barrier. In high-concentrate feeding systems, preventing microbial products from crossing damaged epithelial barriers may be as important as modifying microbial composition itself. Strategies that strengthen rumen or intestinal epithelial integrity, reduce oxidative stress, or limit LPS-induced inflammatory signalling could theoretically interrupt the transfer of ruminal dysbiosis into systemic inflammation [9,65]. However, LPS-neutralizing or barrier-enhancing additives still require stronger in vivo validation in high-yielding dairy cows before they can be recommended as precision tools.

### 6.5. Functional Metabolites and Rumen Epithelial VFA Absorption as Emerging Targets

Compared with broad manipulation of microbial abundance, metabolite-oriented regulation may provide more precise and actionable intervention points because metabolites are closer to host physiological targets than taxonomic markers. Existing evidence on ruminal 3-indoleacetic acid, microbial alanine, and hindgut-derived acetate suggests that key microbial metabolites can participate in inflammatory regulation, glucogenic supply, and hepatic lipid metabolism, respectively [20,22,42]. Rumen epithelial VFA absorption may become an important target for microbiome-oriented precision regulation, but current evidence is still largely mechanistic. Findings from goat SARA models suggest that microbial thiamine-related pathways can enhance epithelial proliferation and VFA absorption [24], providing a potential entry point for epithelial-targeted intervention.

Overall, microbiome-oriented precision regulation should move beyond the simple addition of probiotics or buffers. A more effective strategy should integrate dietary transition management, microbial functional restoration, host metabolic support, barrier protection, and epithelial VFA absorption capacity. Such an integrated approach is more likely to rebuild the balance between high milk yield and metabolic health than interventions aimed at a single microbial taxon or nutrient parameter (Table 2).

## 7. Outlook and Challenges

Although rumen microbiome research has rapidly advanced from taxonomic profiling to multi-omics analysis, the field is still in transition from “which microbes are present” to “what functions they perform and how they affect the host.” Future studies should integrate metagenomics, metatranscriptomics, metabolomics, and host phenotypic data to identify functional genes, active metabolic pathways, and microbial metabolites that are directly linked to productivity and metabolic health [74,75]. Rumen microbiome nutriomics involves harnessing omics technologies for enhanced understanding of rumen microbiome functions and ruminant nutrition.

A second priority is to move from association to causality. Many studies have identified microbial taxa or metabolites associated with SARA, ketosis, fatty liver, or milk production traits, but association-based biomarkers cannot automatically be translated into intervention targets. Functional validation through microbial isolation, in vitro fermentation systems, gnotobiotic or transplantation models, and targeted metabolite supplementation will be required to determine whether candidate microbes or metabolites are drivers, consequences, or compensatory responses of disease [23,74]. In this context, rumen culturomics and functional strain screening are particularly important because they can convert sequencing-based discoveries into cultivable probiotic candidates with defined metabolic functions [76,77].

Machine learning and predictive modelling represent another important direction for microbiome-based precision management. Rumen, saliva, fecal, milk, and blood biomarkers may be integrated with feeding behaviour, production records, and physiological indicators to predict individual risk of SARA, ketosis, or poor adaptation during the transition period. Studies showing that SARA susceptibility is reflected in ruminal, salivary, or faecal microbial features, and that the rumen microbiome is associated with postpartum ketosis development, provide a basis for developing early-warning models [21,26,78]. However, such models should be evaluated not only by statistical accuracy, but also by their robustness across farms, diets, breeds, lactation stages, and sampling methods.

The major challenge for future applications is individual variability. Host genotype, parity, lactation stage, dietary history, environmental stress, and baseline microbiota can all influence microbial responses and intervention outcomes [19,48,79]. Therefore, the goal should not be to define a universal “optimal rumen microbiota.” A more realistic objective is to establish risk-stratified and scenario-specific management protocols, in which microbial indicators are interpreted together with diet structure, production level, transition status, and host metabolic phenotype.

Translation from laboratory discovery to farm practice remains another bottleneck. Multi-omics approaches are powerful for mechanism discovery, but their routine use on farms is limited by sampling difficulty, cost, analytical complexity, and the need for rapid interpretation. Future biomarker systems should therefore prioritize low-cost, reproducible, and easily collected samples, such as milk, saliva, feces, or rumen bolus-derived data, while reserving high-resolution multi-omics for model development and mechanistic validation. Ultimately, the success of microbiome-based precision regulation will depend on whether mechanistic insights can be converted into simple decision tools that guide feeding transition, microbial intervention, and metabolic risk management under commercial production conditions.

## 8. Conclusions

High productivity and animal health are not inherently contradictory. The central challenge is whether high nutritional input can be matched by a stable rumen microbial ecosystem and coordinated host metabolic adaptation. When this coordination is maintained, high-yielding dairy ruminants can efficiently convert dietary resources into milk production without excessive metabolic or inflammatory burden. When it fails, the same production strategy may shift from supporting lactation to promoting metabolic instability. The role of the rumen microbiota in this process should therefore be reconsidered. It is no longer sufficient to regard the rumen microbiota merely as a digestive community responsible for feed fermentation. Rather, it functions as a complex metabolic regulatory system that links nutrition, microbial metabolites, epithelial function, immune responses, and energy metabolism. This conceptual shift provides a new basis for understanding why animals exposed to similar diets may differ markedly in productivity, disease susceptibility, and metabolic resilience. Future research should move beyond descriptive microbial profiling and focus on the molecular mechanisms of host–microbe interactions, especially how functional microbes, key metabolites, epithelial responses, and immune-metabolic pathways jointly determine health outcomes. In practice, microbiome-based prediction, risk stratification, and individualized nutritional management will be essential for translating these mechanisms into production systems. Achieving the coordinated development of high yield and long-term health will depend not on maximizing a single nutritional parameter, but on managing the rumen microbiome as a central component of precision dairy production.

## Figures and Tables

**Table 1:** Microbial and metabolite signals involved in productivity support and metabolic disease risk.

Microbial/Metabolite Feature	Main Function	Related Host Response	Species/Model	References
Propionate	Gluconeogenic precursor	Lactose synthesis, milk yield	Dairy cows	[9,46]
Acetate	Milk fat synthesis, hepatic metabolism	Energy supply	Dairy cows/ruminants	[14,38]
Butyrate	Epithelial energy source	Papillae development	Ruminants	[15,23]
Microbial protein	Amino acid supply	Milk protein synthesis	Dairy cows	[39,42]
Lactate	Acid accumulation	pH depression	SARA models	[8,25]
LPS	Inflammatory stimulus	Systemic inflammation	Dairy cows	[9,10]
3-IAA	Anti-inflammatory signal	Restrained Th17/IL-17 response	Dairy goats	[22,24]

SARA, subacute ruminal acidosis; LPS, lipopolysaccharide; 3-IAA, 3-indoleacetic acid.

**Table 2:** Microbiome-oriented precision strategies for balancing high yield and metabolic health.

Strategy	Target	Proposed Mechanism	Key References
Nutritional preconditioning	Host and rumen adaptation	Gradual adaptation to high-energy diets; stabilizes fermentation and microbial ecology	[66]
peNDF/RDS balance	Fermentation kinetics	Balances acid production and buffering capacity, supports fibrolytic microbes	[67]
Yeast probiotics	Lactate and microbial balance	Stimulates lactate-utilizing bacteria; competes with acid makers; stabilizes rumen pH	[68]
Probiotics	Specific rumen pathways	Target lactate accumulation and improve fermentation stability	[69]
Prebiotics/synbiotics	Microbiota support	Promote beneficial bacteria growth and microbial network robustness	[70]
Plant extracts/essential oils	Microbial modulation	Selectively modulate fermentation, reduce harmful taxa, buffer pH	[71]
Rumen-protected nutrients	Host metabolism	Support hepatic lipid metabolism and reduce metabolic stress	[10]
Barrier/anti-inflammatory modulators	Host–microbe interface	Reduce inflammatory signalling and LPS translocation	[67]
Microbiome biomarker prediction models	Early warning/risk stratification	Integrate microbiome data with host phenotypes	[72]
Phage-based or antiviral strategies	Targeted microbial control	Potential precision suppression of harmful microbes	[73]

peNDF, physically effective neutral detergent fibre; RDS, rumen degradable starch; LPS, Lipopolysaccharide.

## Data Availability

No new data were created or analyzed in this study. Data sharing is not applicable to this article.
